# The Antioxidant Role of Soy and Soy Foods in Human Health

**DOI:** 10.3390/antiox9070635

**Published:** 2020-07-18

**Authors:** Gianluca Rizzo

**Affiliations:** Independent Researcher, Via Venezuela 66, 98121 Messina, Italy; gianlucarizzo@email.it; Tel.: +39-3208-976-687

**Keywords:** soy, soybeans, soy foods, antioxidants, reactive oxygen species, cardiovascular diseases, diabetes, cancer

## Abstract

Oxidative stress seems to play a role in many chronic diseases, such as cardiovascular diseases, diabetes, and some cancers. Research is always looking for effective approaches in the prevention and treatment of these pathologies with safe strategies. Given the central role of nutrition, the identification of beneficial healthy foods can be the best key to having a safe and at the same time effective approach. Soy has always aroused great scientific interest but often this attention is galvanized by the interaction with estrogen receptors and related consequences on health. However, soy, soy foods, and soy bioactive substances seem to have antioxidant properties, suggesting their role in quenching reactive oxygen species, although it was frequently mentioned but not studied in depth. The purpose of this review is to summarize the scientific evidence of the antioxidant properties of soy by identifying the human clinical trials available in the literature. A total of 58 manuscripts were individuated through the literature search for the final synthesis. Soy bioactive substances involved in redox processes appear to be multiple and their use seems promising. Other larger clinical trials with adequate standardization and adequate choice of biomarkers will fill the gap currently existing on the suggestive role of soy in antioxidant mechanisms.

## 1. Introduction

Soy (*Glycine max* L.) is a very important food in human nutrition. It represents a popular food in South-East Asian cuisine and its characteristics have attracted the interest of the food industry [1]. Its nutritional properties and biotechnological characteristics are attractive and explain the widespread use in the production of processed foods, especially as analogs and substitutes of meat intended for vegetarian and self-conscious users [2]. The lower incidence of some chronic diseases among populations that traditionally use soy and soy foods worldwide has suggested their role in the prevention of some pathologies [3]. However, the evidence from the literature suggests a discrepancy in the effect between Asian and Western populations whose consumption by the latter is very limited and only recently adopted. For example, there is a limited amount of prospective studies that have assessed the association between soybean and type 2 diabetes mellitus (T2DM) in Western populations [4]. In 2016, Ding and colleagues conducted a prospective pooled analysis based on three large North American cohort studies (Health Professionals Follow-Up Study, First and Second Nurses’ Health Study) about the influence of soy, soy foods, and soy isoflavones consumption on T2DM risk [5]. The analysis included 163,457 subjects with 9185 T2DM documented incident cases. After multivariate adjustments, there was no association between soy food consumption and T2DM risk but there was a significant inverse association between soy isoflavone and risk of T2DM (HR: 0.89, with 0.83 to 0.96 95% CI; *p* = 0.009 for the trend) for highest compared to the lowest quintile of consumption. A significant inverse correlation persists down to the third quintile of soy isoflavone consumption. Other studies in China, Singapore, and Hawaii showed an inverse correlation between soy and T2DM risk [6,7,8] but this was not confirmed in a Japanese study [9].

In 1999, the US Food and Drug Administration (FDA) released a cholesterol-lowering claim about soy protein foods [10], but recently, based on new evidence, a revocation was proposed [11]. In light of this latter hypothesis, Jenkins at al. performed a cumulative meta-analysis including the before-1999 studies used for the original claim and some additional recent trials, finally selecting 46 studies and 2607 participants for the re-analysis [12]. A minimum reduction of 4.0 mg/dL (with −6.7 to −1.3 mg/dL 95% CI; *p* = 0.004) and a maximum reduction of 7.7 mg/dL (with −11.2 to −4.3 mg/dL 95% CI; *p* < 0.00001) for total cholesterol (TC) was observed with a median soy protein intake of 25 g/d. Moreover, a minimum reduction of 4.2 mg/dL (with −6.6 to −1.8 mg/dL 95% CI; *p* = 0.0006) and a maximum reduction of 6.7 mg/dL (with −10.2 to −3.2 mg/dL 95% CI; *p* < 0.0002) for low density lipoprotein cholesterol (LDL-C) was observed. From the previous trials regarding the first claim of the FDA until 2013, the significance never fell below *p* = 0.002. This modest soy effect, if adopted with concomitant synergic dietary interventions, is comparable with a statin-like reduction effect [13]. Likewise, Health Canada has recently approved a soy cholesterol-lowering effect claim [14].

In a dose-response meta-analysis of 23 prospective cohort studies and 330,826 participants, soy consumption was inversely associated with cancer mortality (RR: 0.88, with 0.79 to 0.99 95% CI; *p* = 0.03; I^2^: 47.1%) and CVD mortality (RR: 0.85, with 0.72 to 0.99 95% CI; *p* = 0.04; I^2^: 50.0%) [15]. Besides, soy isoflavone consumption was also associated with all-cause mortality (RR: 0.90, with 0.82 to 0.98 95% CI; *p* = 0.02; I^2^: 0.0%). Soy protein intake was inversely associated with breast cancer mortality (RR: 0.73, with 0.55 to 0.96 95% CI; *p* = 0.02; I^2^: 63.5%) but no association emerged for all-cause or CVD mortality. In the linear dose-response analysis, a 10 mg/d increase in soy isoflavone was associated with lowering the breast cancer mortality risk by 9%, whereas a 5 g/d increase in soy protein intake was associated with a lowering of 12%. In a recent cohort study of 52,795 US women from the Adventist church, the substitution of dairy milk with soy milk was associated with reduced breast cancer risk (HR: 0.68, with 0.55 to 0.85 95% CI; *p* = 0.001) [16]. In a systematic review and meta-analysis of 30 studies and 266,699 multi-ethnic participants, an inverse association between soy consumption and the prostate risk was highlighted (RR: 0.70, with 0.58 to 0.85 95% CI; *p* < 0.001; I^2^: 68.9%) [17]. Unfermented soy foods (but not fermented foods) were also significantly associated with reduced prostate cancer risk (RR: 0.65, with 0.52 to 0.83 95% CI; *p* < 0.001; I^2^: 60.3%). Likewise, genistein and daidzein but not total isoflavone intakes were inversely associated with cancer risk, which implies a wider mechanism of action not limited to isoflavone bioavailability. Moreover, the genistein and daidzein circulating concentrations were not associated with prostate cancer risk.

It has been hypothesized that the effect of soy on chronic diseases depends on specific bioactive substances and primary isoflavones, and that the protective action involves anti-inflammatory, antineoplastic, antiplatelet mechanisms, and, at least in part, antioxidant mechanisms [18,19,20]. The effect of isoflavones on cardiometabolic risk factors could mainly imply mechanisms unrelated to the interaction with the estrogen receptor [21]. Moreover, oxidative stress seems to influence glycemic tolerance [22], CVD [23], and the genesis of neoplastic pathologies [24]. The implication of oxidative stress in non-communicable diseases suggests the possibility of an effective and safe approach for prevention. The use of soy, soy foods, and soy bioactive substances, widely consumed in the world, could provide an economic and easily available solution in a sustainable nutritional paradigm, already widely discussed for preventive purposes. However, there is a need for comprehensive data collection that defines the effectiveness of soy. The purpose of this review is to clarify the antioxidant properties involved in the beneficial effects of soy, soy foods, and soy bioactive substances on health through the identification of clinical trials on humans.

## 2. Methods

Consultation of the databases was carried out through a systematic search method using the PubMed search engine [25] and a Trials Cochrane Library Central search [26], to identify the relevant works based on the specific inclusion criteria listed below.

The following string for the query was used: “(ROS [Title/Abstract] OR antioxidant [Title/Abstract] OR antioxidants [Title/Abstract] OR oxidative [Title/Abstract]) AND (soy [Title/Abstract] OR soyfood [Title/Abstract] OR soybeans [Title/Abstract] soyfoods [Title/Abstract] OR soybean [Title/Abstract] OR “soy food” [Title/Abstract] OR “soy beans” [Title/Abstract] OR “soy foods” [Title/Abstract] OR “soy bean” [Title/Abstract] OR tofu [Title/Abstract] OR tempeh [Title/Abstract] OR natto [Title/Abstract]) ”.

The formatting has been adapted to the search engine used by including the MeSH terms of interest, where available.

The results of the query were limited to articles in English, including human clinical trials investigating the effect of soy, soy foods, or soy bioactive substances on oxidative stress markers as a primary or secondary outcome. No time restrictions have been applied, specifically from inception of the databases up to 19 May 2020. Interventions that used non-soy isoflavones (e.g., from red clover or *Genista tridentata* L.) were excluded. Case reports, case series, and animal studies were excluded. Besides, the reference lists of the manuscripts identified were checked for additional entries.

## 3. Human Clinical Studies: The Effect of Soy on Oxidative Biomarkers

A total of 2360 entries were obtained from PubMed and, after limiting the results to articles in English and on humans, 398 entries were selected. A total of 310 results were obtained from the Cochrane library. Following the removal of 62 duplicates, 646 articles were obtained. After a selection by title and abstract, 51 articles were obtained according to the eligibility criteria. Eleven additional articles were extracted from reference lists after reading the full-text already selected. The full-text of 2 articles were unavailable and 2 articles were duplicates of trials already present among the selected publications for a total of 58 articles used for the final synthesis that are summarized in Table 1.

The number of subjects in trials ranged from 6 to 200 individuals. A total of 22 manuscripts employed North American populations, 11 Iranian, 1 Canadian, 1 Hawaiian, 2 South American, 5 North European, 1 Slovakian, 3 South European, 2 Australian, 1 Indian, and 9 South-East Asian. The design of the trials was heterogeneous: 28 parallel trials, 25 cross-over trials, and 5 single-arm trials. Of these, 40 double-blind RCTs, 7 single-blind RCTs, 7 open-label trials, 1 non-randomized trial, and 3 that were not well-defined RCTs regarding the blinding.

Twenty articles were based on healthy individuals and 20 articles were based on metabolic pathologies, such as diabetes, dyslipidemia, hypercholesterolemia, hypertension or obesity, and simple overweight. Moreover, 2 articles were based on gestational diabetes, 2 on PCOS, 1 on nephropathy, 2 on neuropathic T2DM, and 1 on diabetic retinopathy. Ten articles were based on menopausal healthy populations. The intervention time ranged from 60 post-prandial minutes after single soy ingestion to 12 months of intervention. Selected trials were carried out using soy and soy foods (including traditional, fermented and non-fermented foods, as well as texturized soy) or isolated components, such as isoflavones, oils, sterols, proteins, or oligosaccharides. Two studies used soybeans while 18 manuscripts used soy foods, such as soy drink, tofu, and soy flour, with one manuscript using various soy foods, including soy nuts. Moreover, soy nuts were exclusively used in 4 trials. Three trials used fermented foods, i.e., Doenjang [39], natto [81], and dark soy sauce [62]. Eleven manuscripts used soy protein isolates with associated isoflavones. One manuscript used a mix of proteins and sitosterol [40], 1 black soy peptides [61], and 1 used pasta enriched with soy germ [41]. Eleven manuscripts evaluated the effect of isolated soy isoflavones on oxidative stress markers. One manuscript used a mix of isoflavones and oligosaccharides [70] and 1 oligosaccharide only [45]. Five manuscripts evaluated the use of texturized soybean bars or burgers. Finally, 2 articles assessed the effect of soybean oil [42,83].

Taking into account interventions where concentrations of isoflavones were declared, the daily intake varied from 17.13 mg/day [48] to 207 mg/day [69]. The intake of isoflavones in interventions with soybean oil and oligosaccharides was negligible [42,45,61,83]. The intake of soy protein in selected trials ranged from 8 g/day [40] to 52 g/day [59].

Regarding the oxidative biomarkers, different techniques and molecules have been used which are summarized in Table 2.

A total of 44 articles used markers based on lipid oxidation (TBARS, PGF2α, LARC, LOOH, and Ox-LDL); 21 articles used indicators of total antioxidant capacity (FRAP, ABTS, TAP, AOPP, ORAC, and whole plasma oxidation); 19 articles assessed the levels of endogenous oxidation biomarkers, such as molecules and enzymes involved in antioxidant defenses or response to oxidative stress (SOD, GPx, GSH, GSSG, GSR, CAT, MPO, COX-2, PON, Nrf-2, and uric acid); 5 articles used nucleic acid oxidation markers (8-OHdG, 5-OHmdU, and the comet assay); and 2 articles used protein carbonyl oxidation markers. About articles with multiple markers usage, no manuscript evaluated at least one of each class of markers listed above (nucleic acids, proteins, fats, endogenous oxidation biomarkers, and total antioxidant capacity) at the same time.

To better summarize the results that emerged from the selected articles, a subdivision was made based on some features of the interventions, evaluating the results based on the type of biomarkers adopted.

### 3.1. The Effect of Soy, Soy Foods, and Soy Bioactive Molecules on Oxidative Stress

#### 3.1.1. Soy Foods

Of the 18 trials that used soy foods, mixed results were highlighted. The DNA oxidative damage markers showed an improvement effect on the intervention [75], partial effect [66], or no statistically significant effect [51]. Regarding fat oxidation, an improvement effect has been detected [27,28,58], as well as partial [47,69], slightly worsening [69,77], and no statistically significant effect [64,65,75,86]. About endogenous oxidation biomarkers, 1 work showed improvement of markers [74], two works showed mixed results [47,65], and three works did not detect significant variations [32,33,51]. Regarding the markers of total antioxidant capacity, 3 papers did not show a significant effect after the consumption of soy foods [65,66,71], while 3 papers showed improvement in the antioxidant capacity [48,74,78]. No protein oxidation markers were used by the selected trials.

The two trials by Beavers and colleagues [32,33] have been conducted with the same protocol on the same subjects. The two works differed from the presence or not of an exercise bout in the trial protocol. However, the results of the oxidation markers were not significant in both cases. In the work of Miraghajani and colleagues [65], while a partial response on endogenous oxidation biomarkers in both arms was noted, the trial was designed to detect the effect of taking soy milk with probiotic characteristics compared to normal soy milk. An improvement has been achieved compared to non-probiotic soy control, but with the lack of a soy-free placebo arm. A similar design was used in the clinical trial of Hariri and colleagues [51] but with improvements only with probiotic milk and not attributable to soy, because it was not significant in the control group with unfermented soy (effect over time compared to baseline). Zemel and colleagues conducted a clinical trial in which the soy phase was considered as a control [86]. In this circumstance, an improvement effect of the antioxidant capacities was detected but there was no significant variation during the soy phase. As in other works cited above [51,65], Ahn-Jarvis and colleagues did not use soy-free control, being the two phases of intervention based on soy bread or soy drinks with a similar intake of isoflavones [27]. The ameliorative effect on oxidative stress was obtained with the soy drink alone, demonstrating the role of the food matrix on oxidative stress.

Overall, the effect of soy foods seems unconvincing but it is unlikely that the oxidation worsened after soy intake.

Rayan-Brochers and colleagues conducted a clinical trial with three parallel arms that included an intervention with soy milk, one with cow’s milk (as placebo) and one with cow’s milk added with 70 mg of isoflavones, the same amount obtained from the intervention with soy milk [75]. The outcomes of the two arms of intervention on the oxidation markers were similar, suggesting that the antioxidant effect was borne by isoflavones and not by other components of soy milk. Similarly, in the work of Nhan and colleagues, the control phase of the trial consisted of a soy drink with a low isoflavone content and, although there were no significant differences between the phases of the trial, a correlation emerged between the variations of the markers of oxidative stress and the daily dose of isoflavones [69]. The presence of a biphasic curve has been hypothesized, based on reduced urinary excretion of PGF2α for the intakes of daidzein <60 mg/d but with an increase in the excretion of PGF2α for daily intakes >72 mg.

#### 3.1.2. Pure Isoflavones

Although isoflavones are likely to play a role in the antioxidant properties of soy, the effect of soy foods could depend only in part by the isoflavones’ action. Eleven manuscripts used isolated soy isoflavones. Regarding the possible damage of nucleic acids, 2 manuscripts have shown improvement through the intake of isoflavones [43,75]. About the oxidative stress on fatty acids, 3 manuscripts showed an improvement [56,63,72], while 6 manuscripts showed no significant variations [34,43,50,67,68,75], with 1 manuscript showing a partial effect [46]. About endogenous oxidation biomarkers, an improvement was found after the intake of isoflavones in some cases [39,56,63] but not significant in other cases [34,67]. Regarding the effect on total antioxidant capacity, no clinical trial has shown improvement of the markers used [50,56]. No manuscript has evaluated the oxidation status of the proteins following the intake of soy isoflavones.

Overall, as in the case of the soy foods previously evaluated, there does not seem to be a clear protective effect of isolated isoflavones on oxidative stress. However, in Li and colleagues’ work, based on a 4-week double-blind RCT on 100 patients with IHD versus 100 placeboes (the largest population sample among selected trials), all four oxidation markers (endogenous and lipid biomarkers) improved after the intervention with soy isoflavones [63].

In the work of Oh and colleagues, 2 g/day of soy polysaccharides combined with isoflavones (about 155 mg/day) caused an increase in GPx but did not influence the CAT and PON levels [70]. In this context, it was not possible to discriminate between the effect of the isoflavones and that of the soy polysaccharides.

#### 3.1.3. Proteins

Results on lipid oxidation markers following intervention with isolated soy proteins showed improvement [29,30,54,57], as well as partial results [59], pejorative [84], or no significant results [36,44,53,79,84]. About the oxidation of proteins, 1 work found no significant effects [44] and 1 showed partial effects [84]. The effects on total antioxidant capacity were ameliorative [29,30,84], partial [80], or not significant [53]. No intervention trials with isolated soy proteins used markers for the oxidation of nucleic acids or the evaluation of endogenous oxidation biomarkers. In the clinical trial of Campbell and colleagues, there was not any change in biomarkers, although it was a post-prandial evaluation after 6 h after a single-meal soy ingestion [36]. Similarly, Heneman and colleagues evaluated oxidative biomarkers after 8 h post-prandial of a single intake of soy protein, without any significant effect in presence or not of isoflavones with soy protein [53]. Furthermore, in the work of Engelman and colleagues [44] and the work of Steinberg and colleagues [79], the effect of soy protein, with or without isoflavones, showed no significant differences in biomarkers. In the work of Swain and colleagues, the effect of isolated soy proteins containing isoflavones did not show any significant differences compared to soy proteins without isoflavones, with an antioxidant effect correlated to soy intake and not to isoflavones [80]. Similarly, in the clinical trial of Jenkins and colleagues, the antioxidant effect seems to refer to the soy supply and not to the isoflavone content [59]. In the work of Vega-López and colleagues [84], the antioxidant effect was independent of the content of the isoflavones but in the case of the evaluation of MDA levels, the pejorative effect had been observed only in the arm with isoflavones.

The antioxidant effect of soy in the form of protein isolates does not suggest a clear protective outcome. From the works comparing a soy protein isolate with or without isoflavone, it seems that the latter is not decisive for obtaining the antioxidant effect. The only negative effect that emerged seems to be related to isoflavones and not to soybeans [84].

Black soy is a variant of soy known to have particularly high levels of phytochemicals with promising applications on human health [103]. In the work of Kwak and coworkers, black soybean peptides were used against a placebo (casein) to evaluate their antioxidant ability [61]. The improvement in lipid oxidation and the increase in SOD levels were significant in the intervention arm.

In Cicero and colleagues’ work, 8 g per day of soy protein with beta-sitosterol showed no improvement in the lipid oxidation status compared to the baseline in an open-label single-arm trial [40]. Clerici and colleagues showed the improvement of lipid oxidation markers, total antioxidant capacity, and increased GSH concentration using soy-germ enriched pasta containing 31–33 mg/day of aglycone isoflavones [41]. It was not possible to discriminate between the effect of proteins or other phytochemicals contained in the soy-germ compared to the isoflavones.

#### 3.1.4. Nuts

Among the 5 manuscripts that used soy nuts, 3 trials showed an improvement on lipid oxidation markers [29,30,73] and 3 trials showed an improvement effect on the total oxidation capacity [29,30,76]. No selected manuscript showed negative effects on any marker, mixed-effects, or no significant effects. None of the articles evaluated the DNA oxidation, protein oxidation, or endogenous oxidation biomarkers. The use of soy nuts is very common in the world and represents one of the foods with the highest concentration of isoflavones [73].

The work of Azabakht and colleagues employed two different soy-based interventions with isolated proteins or soy nuts [29]. The improvement in total antioxidant capacity was significant compared to the control for both soybean interventions but higher in the soy nuts group. While, regarding lipid oxidation, there was no difference between the two interventions with soy. Even if the intake of phytoestrogens in the soy nuts arm was slightly greater, a beneficial effect of the food matrix is not excluded. Even in the work of Bakhtiari and colleagues [30], there were no differences in the effect on the oxidation markers between the soy protein arm and the soy nut arm, although the latter had higher isoflavones levels, as highlighted in the work of Azabakht and colleagues [29]. In the work of Sen and colleagues already cited, various soy-based foods, including soy nuts, were used, but the choice of the types of foods was a participants’ option, with a total daily intake of 50 mg of isoflavones [77]. There was, therefore, no sub-analysis for the specific effect concerning a single food used.

The limited number of trials using soy nuts that emerged from the selection does not allow solid conclusions to be drawn. However, albeit limited, an improvement in lipid oxidation and total antioxidant capacity were shown. The use of soy nuts is very promising for its widespread use in the world, the high content of isoflavones, and the minimum amount of transformation to obtain the final product. The comparison between soy proteins containing phytoestrogens and soy nuts seems to show an overlapping or slightly unbalanced effect towards soy nuts and this could be further explored.

#### 3.1.5. Snack and Texturized Soy Foods

Opposed to traditional soy foods, there are foods obtained through transformation techniques that lead to the change of the texture and taste of soy through high-pressure and high-temperature procedures. These lead to the formation of soy matter useful for the production of snacks, burgers, and other meat substitutes. Generally, the soy from these processes undergoes a profound transformation that is thought to have a negative effect on the bioavailability of isoflavones [1]. Five trials using snacks or textured soy proteins were selected. Three manuscripts showed an improvement in lipid markers [55,60,82] while 1 manuscript showed mixed results [85]. Two articles showed improvement of endogenous oxidation biomarkers [35,60] while 1 showed no significant variation [55]. One manuscript showed improvement in total antioxidant capacity [35] while 2 manuscripts did not reach significance [55,60]. No manuscript adopted markers for the oxidation of proteins or nucleic acids. No manuscript showed worsening outcomes after soy interventions. Overall, the results suggest an ameliorative effect on oxidation by snack or soy-textured products, although the number of trials is still limited, and not all of them have shown significant results. Since this type of highly processed food has often a reduced concentration of bioavailable isoflavones, the antioxidant effect could partly depend on other components. No trial indicated isoflavone content in aglycone equivalents. However, in 1 trial the control was obtained through the alcoholic extraction of isoflavones, still indicating a role for these bioactive substances in the antioxidant effect [85]. Nevertheless, there was no evaluation of the effect from the baseline; so, it was not possible to evaluate the contribution of other soy components.

#### 3.1.6. Fermented Soy Foods

Fermentation seems to favor the breakdown of the glycosidic bond of the isoflavones and therefore the transformation into the aglycone form with consequent increases in the bioavailability of the isoflavones thanks to the greater diffusion capacity of these compounds through the intestinal mucosal barrier [104]. Among the 3 trials identified, 1 trial showed an improvement in lipid oxidation [81], 1 showed mixed results [62], 1 trial showed enhanced total antioxidant capacity and endogenous oxidation biomarkers [38], while 1 work did not show significant effects on DNA oxidation [62]. No selected manuscripts used markers of protein oxidation. Even if none of the works showed negative results, the number of trials that emerged does not allow definitive conclusions to be drawn. Fermented soybeans are considered highly bioavailable foods rich in isoflavones and other bioactive compounds thanks to the action of microorganisms. Other works will help to understand if this aspect represents an advantage for the antioxidant effect of fermented soy foods. In the trial of Taniguchi-Fukatsu and colleagues, although the content of isoflavones was not declared, the placebo phase consisted of boiled, unfermented soybeans [81]. The improvement of the oxidation biomarkers indicated that the effect depended on the modification of soy during fermentation. It needs to be clarified if it was a matrix effect, a greater bioavailability of soy phytochemicals after transformation, or depending on other molecules or substances of microbial origin released by the starter microorganisms.

#### 3.1.7. Soybeans

Only 2 papers used soybeans in the intervention arm of the selected clinical trials. Hamatdar and colleagues did not show any significant differences in lipid oxidation status using three cups of soybeans per week [52]. However, the intake could be insufficient to trigger an effect. Celec and colleagues showed improvement in lipid oxidation and total antioxidant capacity with 2 g of soybeans per kg of body weight per day [37]. Some discrepancies have been identified in the gender sub-analysis, with the loss of significance on total antioxidant capacity among men.

#### 3.1.8. Soybean Oil

From the selected manuscripts, two trials used soybean oil. Soybean oil does not contain isoflavones due to the extractive procedures during production that does not allow the recovery of these substances [2]. In the clinical trial of Costa and Silvia et al., the total antioxidant capacity improved in the soybean oil arm (control) compared to the intervention with Brazilian walnut oil [42]. However, a worsening effect was shown on lipid oxidation. The worsening of the lipid oxidation biomarkers found would depend on the greater concentration of polyunsaturated fatty acids in soybean oil, easily prone to peroxidation. Conversely, the Brazilian walnut oil had a lower concentration of polyunsaturated fatty acids and involved a greater supply of saturated fatty acids, less prone to peroxidation. Similarly, in the work of Utarwuthipong and colleagues, the soybean oil arm showed a reduced lag time of LDL oxidation compared to soybean oil and/or rice oil, demonstrating the abovementioned hypothesis of greater susceptibility [83].

### 3.2. The Effect of Soy on Exercise-Born Oxidative stress

Five articles assessed the effect of soy on oxidation markers in conjunction with an exercise session [33,35,39,48,54,74]. The exercise-born oxidative stress is a phenomenon known and studied also for the possible implications in skeletal muscle adaptation [105]. The soybean intake seems to improve the total antioxidant capacity [35,48,74] and the oxidation status of the fatty acids, although derived from just one trial [54]. Endogenous oxidation biomarkers also seem to improve [35,39,74], although one work showed no significant results [33]. None of the identified works showed outcomes on markers of protein or nucleic acid oxidation. None of the selected works showed worsening effects following soy intake. It is very difficult to give a conclusive statement, based on the great heterogeneity of the experimental setting, such as time, type, and intensity of exercise.

### 3.3. Soy Postprandial Antioxidant Effect

Five articles assessed the effect of soy from 1 to 8 h in the postprandial period [36,50,53,62,78]. Among the trials selected, only 1 article showed partially reduced lipid oxidation after soy intake [62] and another one showed improvement in total antioxidant capacity [78]. In the remaining trials, there was a lack of significance for the antioxidant effect on lipids, the total antioxidant capacity, and oxidation of nucleic acids. Maybe, the limited time elapsed between the intake and the assessment was too short to have a clear effect, but currently, it does not seem that soy improves the redox status in the postprandial period after its ingestion.

### 3.4. The Soy Antioxidant Effect in Menopause

Eleven articles recruited healthy individuals in the perimenopausal phase [32,33,34,44,48,67,68,72,75,79,80]. Menopause is a period of hormonal changes in women in which the risk of metabolic pathologies increases [106]. Among the selected trials, the work of Swain et al. showed a correlation between total antioxidant capacity and soy intake, independent of the isoflavone content [80], while Pusparini and colleagues showed an improvement in the oxidative status of lipids following the intake of soy isoflavones [72]. In the double-blind, parallel RCT of Ryan-Borchers et al., the reduction of nucleic acid oxidation was similar in the arm with soy milk compared with cow’s milk added with soy isoflavones [75]. All the other works showed no significant improvement in the oxidation markers after soy intake.

### 3.5. Bioavailability and Metabolism Od Soy Isoflavones

Although it is not possible to draw definitive conclusions, some clinical trials suggest a role for isoflavones in the soy antioxidant effect. For this purpose, it is useful to understand the absorption of isoflavones through the evaluation of serum or urinary levels of both isoflavones and metabolites, such as equol, *O*-desmethylangolensin (*O*-DMA), 6-hydroxy-*O*-desmethylangolensin (6-OH-*O*-DMA), dihydrodaidzein (DHD), and dihydrogenistein. Among the manuscripts selected, 19 trials evaluated the levels of isoflavones and related metabolites [27,28,29,37,39,41,43,53,57,59,66,69,73,75,77,79,82,84,85]. In general, although with a wide individual variability, the soybean intake lead to an increase in blood [28,29,37,39,41,43,53,66,73,75,79,82,84,85] or urinary isoflavones [57,59], but not in all trials [69]. The levels of metabolites after soy treatment were also higher in plasma [79,85] and urine [59]. In the clinical trial of Steinberg and colleagues, although the number of equol-producers (individuals capable of metabolizing daidzein into equol) was not sufficient to assess a statistical significance, the inclusion of only these subjects in the sub-group analysis (10 out of 28 individuals) did not change the results of oxidative biomarkers that remained not significant [79]. In the cross-over trial of Sen et al., the equol-producers were equally distributed between the groups at baseline [77]. A significant positive correlation was shown between the urinary concentrations of isoflavones and the level of urinary isoprostane; it was persistent after the stratification by the equol-producer but lost significance for the subjects who were not equol-producers. In the manuscript of Mitchell and colleagues, 1 out of the 4 participants in the intervention group was an equol-producer [66]. However, no sub-analysis was possible for obvious numerical reasons. A gender-specific variability in gut microbial competence for isoflavones metabolism was proposed [107]; however, Celec and colleagues, although they have found differences in oxidative response between men and women, did not investigate isoflavone metabolism [37]. Ahn-Jarvis and colleagues highlighted that their cohort was represented by 30% equol-producers and all but three subjects were *O*-DMA and DHD producers. They showed a gender difference in the excretion of isoflavone metabolites, greater in women than in men after taking soy bread [27]. Despite these differences, no gender variability in the excretion of isoflavones was highlighted following the intake of soy drink, despite a comparable intake of aglycone isoflavones in the two interventions. Increased production of equol, *O*-DMA, and DHD was shown in the soy bread arm compared to soy beverage with concomitant lower urine concentrations of daidzein, but only in women. Similarly, Ryan-Borchers and colleagues showed differences in the absorption and metabolization of isoflavones when taking soy drink or isolated isoflavones added to cow’s milk [75]. While the circulating genistein levels were more elevated in the intervention arm with isolated isoflavones than in the soy drink, equol concentrations increased in the soy drink arm while they did not show significant changes in the arm with isolated isoflavones. Furthermore, urinary genistein and daidzein were higher in the arm with isoflavones but the equol excretion was greater in the arm with soy drink. Despite these differences, there were no different outcomes between the two interventions. This suggests an effect of the matrix or other components on the metabolizing capacity of soy isoflavones, which is not necessarily complementary to the absorption capacity. In the trial of Reverri and colleagues, 47% of the participants were equol-producers while 71% were *O*-DMA producers [73]. However, the ability to produce isoflavone metabolites was not related to oxidative stress or other metabolic markers in the linear mixed model. Nhan et al. found a statistically significant positive association between the combined urinary excretion of daidzein, genistein, and equol, as well as change in urinary PGF2α [69]. However, only 2 out of 8 participants were equol-producers so it was not possible to highlight a clear effect based on the ability to metabolize isoflavones.

## 4. Oxidative Stress Implication in Human Health: Pathophysiological and Cellular Mechanisms

Traditionally, oxidative stress has been implicated in mechanisms of aging through the production of free radicals with potentially harmful effects on cellular structures [108]. The oxidation of proteins, nucleic acids, and lipids leads to an increase in the risk of various pathologies, including cancer [109]. Similarly, oxidative stress is recognized as a major contributor to the initiation and progression of cardiovascular dysfunctions [110,111] and atherosclerosis [112]. Among the various mechanisms involved, ROS play a role in angiogenesis, causing cell apoptosis of endothelial cells and increasing the adhesive capacity of monocytes [23]. Moreover, the increase in visceral fat promotes the release of inflammatory cytokines, which, in turn, are responsible for the generation of the oxidative stress implicated in thrombogenesis and atherogenesis [113]. Chronic hyperglycemia has been proposed as a cause of mitochondrial and extramitochondrial oxidative stress [114]. In particular, mitochondria cope with the oxidizing action of oxygen during electron leakage [115]. Oxidative stress appears to affect glucose tolerance, as hypothesized following cell culture studies, suggesting that oxidative stress impairs insulin-mediated translocation of GLUT4 [116] and suppressing the gene expression of insulin in pancreatic β-cells [117]. ROS generated from glucose metabolism act as signals for insulin secretion [118], so there is an equilibrium between the formation and removal of intracellular ROS acting on glycolysis and the Krebs pathways by uncoupling the stimulus-secretion mechanisms that impair insulin secretion [119]. The pathogenesis of diabetes mellitus has been linked to oxidative and inflammatory mechanisms that appear to be responsible for the long-term consequences of diabetes [120].

Oxidative stress represents a possible mechanism in dyslipidemia and hyperglycemia through the reduction of the volume of beta cells of the pancreatic Langerhans’ islets [121]. The same vascular effect mentioned above could explain, at least in part, the micro and macrovascular complications typical of diabetes [122]. For these reasons, potential approaches to diabetes have been proposed through the use of antioxidants [123,124,125]. For example, the synergic treatment of rosiglitazone and metformin improves vascular function through the reduction of inflammation and oxidative stress [126]. Even in secondary prevention, mitigating oxidative stress was proposed as an approach for diabetes, as well as for associated cardiovascular diseases [127,128]. Oxidative stress can also induce modification of nucleic acids, such as breaking of the scaffold of DNA and other epigenetic modifications, e.g., increased methylation, with consequent effects on gene expression and therefore acting on the pathogenesis of cancer [129,130].

Over the years, the literature on soy and its potential health effects have focused primarily on soy proteins and the associated isoflavones. Phytoestrogens could manifest their antioxidant effect thanks to their ability to donate hydrogen atoms to free radicals, manifesting a quenching action on oxidative stress [131]. However, genistein and daidzein likely have a lower antioxidant capacity than quercetin, due to the presence of only one hydroxyl group compared to the two hydroxyl groups in the B-ring of the latter [132]. In cell cultures, the action of soy isoflavones increases the expression of the metallothioneins and antioxidant enzymes, thus also showing an indirect antioxidant effect [133,134]. Another indirect effect on oxidative stress could be represented by the ability of soy isoflavones to modulate the cellular management of iron by removing molecules that can trigger Fenton reactions in the cytosol [135]. Absorption and metabolization seem to be crucial aspects for the efficacy of isoflavones and explain the frequent discrepancies between in vitro and in vivo studies [136,137]. Despite the great interest, the biological activity of soy is not limited to proteins and phytoestrogens but also other functional components, such as peptides, saponins, phytosterols, protease inhibitors, and phytic acid [138,139]. Recently, research has shown great interest in bioactive soybean peptides, such as lunasin, BowmanBirk inhibitor, and soymorphins, especially for their ACE-inhibitor-like efficacy as an effective approach in the fight against hypertension and cardiovascular diseases [138]. Bioactive peptides are 2- to 20-amino-acid-long molecules that are activated only after being detached from the parent protein by food transformation or intestinal digestion processes [140]. Lunasin and soy protein seem to play a ROS scavenger action [141,142]. Many amino acids seem to contribute to the antioxidant effect of peptides [143]. Since the fermentation of foods seems to cut the protein components of soy, the antioxidant effect also increases in comparison with unfermented foods [144]. Another class of bioactive compounds, of which soy is the main source, are saponins [145]. The antioxidant capacity of these compounds is far greater than that of SOD [146]. However, the effectiveness of these compounds depends on their bioavailability. These compounds in food are found in the glycoside form and therefore not bioavailable until the oligosaccharide moieties are cut to obtain the aglycone forms, called soyasapogenols [139]. As in the case of isoflavones, saponins are not naturally found in the aglycone form but can be obtained following the processing of soy foods [139]. Although saponins are generally considered to be poorly bioavailable, gut bacteria can hydrolyze oligosaccharides moieties [147]; however, saponins or their metabolites were not recovered in the urine of healthy women after intake [148]. Other components present in soybeans, although in limited quantities, are phytosterols and triterpenes. These compounds are mainly studied for their cholesterol-lowering action, which does not seem to require intestinal absorption to exert their action, being generally poorly absorbed and not requiring the simultaneous presence of cholesterol in the intestinal lumen [139]. The mechanism of action is still unclear but an antineoplastic effect of phytosterols has been proposed through an antioxidant effect [149]. Other phytoestrogens different from isoflavones are present in soy, mainly characterized in other plant foods such as lignans [150,151]. As with other bioactive compounds discussed, the absorption of lignans is closely linked to the ability to metabolize the glycoside form by the intestinal microbiota, capable of transforming them into enterolignans [152]. Although not specifically shown for soy lignans, an antioxidant and cytoprotective effect has been shown, based on the ability of lignans to scavenge free radicals [153,154,155,156,157]. Phytic acid, a molecule responsible for the accumulation of phosphorus in the plant, is also capable of exerting an antioxidant effect through its ability to chelate metal ions [156,157]. However, phytic acid may not be effectively absorbed from the intestinal lumen [158]. In addition to isoflavones and lignans, other polyphenols can be found in soybeans, including phenolic acids, anthocyanins, tannins, and stilbenes, with strong antioxidant power. These components are often associated with external layers of soybean and with a wide variability, depending on the cultivars and processing [103].

## 5. Remarks and Future Perspectives

Literature shows an evident gap regarding the antioxidant mechanisms of soy in health. This discrepancy of the literature is particularly relevant taking into consideration that the antioxidant effect of soy is frequently cited but not described in depth. The causes of this phenomenon are manifold: (i) The interest is largely focused on the interaction between isoflavones and the estrogen receptors. Even if this effect is the most evident, the currently available evidence suggests that it is not sufficient to justify the effects of soy on health. Furthermore, isoflavones itself can act with multiple mechanisms, probably concerted with non-isoflavone soy phytochemicals. (ii) There are many markers of oxidative stress but there is no consensus of methods and cutoffs adequate to detect the antioxidant ability of a substance in vivo. (iii) The presence of numerous confounding factors, especially dietary ones, raises critical issues in detecting the real effects of soy against oxidation. This has frequently led to the use of animal models whose data are difficult to transfer to humans for physiological differences. For example, rodent and non-human primate differently metabolize isoflavones. Moreover, there are discrepancies between experimental animal conditions and human real life. (iv) The detection of a physiological effect could be greatly different by the type of food used (soybeans, traditional soy foods, and soy-based meat analogs and supplements) and by the population reference (healthy individuals, patients with metabolic diseases, pre or postmenopausal, athletes, etc.).

## 6. Conclusions

Overall, the antioxidant effect seems to play an important role in the beneficial effects of soy and its bioactive substances in human health. More in-depth studies are needed solve inconsistencies in the literature. There is a need for clinical trials that simultaneously evaluate the antioxidant effect of soy on the main groups of markers available (i.e., on nucleic acids, proteins, fats, total antioxidant capacity, and endogenous enzymes). It is necessary to evaluate these aspects with adequate time laps to avoid interpreting transitory effects. Furthermore, the use of large populations would allow a correct assessment of the presence of equol-compliant individuals that could, at least in part, clarify some of the discordant data present in the literature.

## Figures and Tables

**Table 1:** Human clinical studies of the antioxidant effect of soy.

Reference	Design	Intervention	Biomarkers	Subjects	Nationality	Outcomes
Ahn-Jarvis et al. 2012 [27]	Cross-over RCT	Soy bread (20 g protein99 mg aglycone isoflavones) or soy beverage (93 mg aglycone isoflavones) per day for 3 weeks each.	Ox-LDL	20 adult subjects with hypercholesterolemia	North-American	Lower LDL oxidation in beverage intervention. Lag time after bread intervention was not significant.
Ashton et al. 2000 [28]	Cross-over RCT	290 g/day tofu (119.8 mg isoflavones) vs. 150 g lean meat for 1 month each.	Ox-LDL	42 healthy men	Australian	Lower LDL oxidation.
Azadbakht et al. 2007 [29]	Cross-over RCT	30 g/day of soy protein (84 mg phytoestrogen), soy nut (102 mg phytoestrogen) or DASH (control) for 8 weeks each.	TBARS, FRAP	42 postmenopausal women with metabolic syndrome	Iranian	Improvement of oxidative biomarkers in soy phases.
Bakhtiari et al. 2019, 2011 [30,31]	Single-blind RCT	35 g/day soy nut (117.2 mg isoflavones), or 35 g/day soy protein (96.2 mg isoflavones) vs. control for 12 weeks.	FRAP, TBARS	25 per arm for old women with metabolic syndrome	Iranian	Improved oxidative biomarkers compared to control and before the intervention. No significant differences between soy interventions.
Beavers et al. 2009 [32,33]	Single-blind RCT	3 servings/day of Vanilla soy beverage (90 mg isoflavones) vs. dairy (control) for 4 weeks (with or without exercise bout in different publications).	SOD, GPx, COX-2	Healthy postmenopausal women: 16 treated, 15 control	North-American	Not significant.
Brandão et al. 2019 [34]	Double-Blind RCT	80 mg/day isoflavones vs. placebo for 4 months.	SOD, CAT, GPx, TBARS	Postmenopausal women with insomnia: 19 treated, 19 placebo	South-American	Not significant.
Brown et al. 2004 [35]	Double-Blind RCT	3 soy bars/day (33 g protein) vs. whey bars (placebo) or control for 9-weeks strength training protocol.	ABTS, MPO	Young men experienced weightlifters: 9 soy, 9 placebo, 9 control	North-American	Preserved antioxidant capacity.
Campbell et al. 2006 [36]	Cross-over RCT	39 g soy protein shake single intake (85 mg aglycone isoflavones) vs. 40 g milk protein (placebo) with 6h post-prandial evaluation each.	Ox-LDL	50 healthy men	North-American	Not significant.
Celec et al. 2013 [37]	Single-arm, open-label trial	2 g/kg BW/day soybeans for 1 week.	TBARS, AOPP, ABTS	88 young healthy adults	Slovakian	Improvement of oxidative biomarkers with gender discrepancies (AOPP not significant in men).
Cha et al. 2014 [38]	Double-blind RCT	9.8 g/day of Doenjang supplement vs. placebo pills for 12 weeks.	ORAC, CAT, TRAP	Overweight subjects: 26 treated, 25 placebo	Korean	Increased antioxidant biomarkers.
Chen et al. 2004 [39]	Double-blind RCT	150 mg/day isoflavones vs. placebo for 4 weeks before two sessions of exercise bout at 80% VO2max.	SOD, CAT, GPx	30 healthy men: 15 treatment, 15 placebo	North-American	Higher pre-exercise SOD attenuated post-exercise CAT and GPx exercise-induced increment.
Cicero et al. 2004 [40]	Single-arm, open-label trial	8 g/day soy protein with 2g/day β-sitosterol for 40 days.	Ox-LDL Ab	36 moderately hypercholesterolemic subjects	Italian	Not significant.
Clerici et al. 2011 [41]	Cross-over RCT	80 g/day soy germ enriched pasta (31-33 mg/day aglycone isoflavones) vs. conventional pasta (placebo) for 8 weeks each.	PGF2α, FRAP, GSH, Ox-LDL	26 T2DM patients	Italian	All markers improved in soy phase.
Costa e Silvia et al. 2020 [42]	Double-blind RCT	10 mL/day Brazil nut oil vs soy oil (control) for 30 days.	TBARS, ABTS	31 adult patients with metabolic syndrome (15 BNO and 16 SO)	South-American	MDA decreased in BNO group while TEAC increased in SO group
Djuric et al. 2001 [43]	Single-arm, open-label trial	50 mg/day isoflavones (women), 100 mg/day isoflavones (men) for 3 weeks.	5-OHmdU, PGF2α	20 healthy subjects	North-American	Reduced DNA oxidative damage without significant lipid oxidation changes.
Engelman et al. 2005 [44]	Double-blind RCT	40 g/day of soy protein isolate with or without isoflavones, with or without phytate for 6 weeks.	Ox-LDL, plasma protein carbonyls, PGF2α	55 postmenopausal women (14 w/o I and F; 13 w/o I and w F; 14 w I and w/o F; 14 w I and F)	North-American	Not significant.
Fei et al. 2014 [45]	RCT	10 g/day Soybean oligosaccharides vs. control for 8 weeks.	SOD, GPx, CAT, TBARS	Pregnant women with GDM: 46 treatment, 51 control	Chinese	Improvement of oxidative biomarkers.
Fritz et al. 2003 [46]	Cross-over RCT	2.01 (high), 1.01 (low), or 0.15 (control) mg/Kg BW/day isoflavones for 12 weeks each.	Polar and non-polar LARC	10 healthy premenopausal women	North-American	Urinary non-polar LARC levels were lower in isoflavone diets than control. Polar compounds were not significantly different.
Gardner-Thorpe et al. 2003 [47]	Cross-over RCT	3 scones/day (soy flour-based, 120 mg isoflavones) or wheat-based (placebo) for 6 weeks each.	FOX 1, Ox-LDL (Cu or MPO)	20 healthy men	Irish	Improvement of MPO Lag time and FOX 1 without any changes in Ox-LDL with Cu.
Hanachi et al. 2007,2008 [48,49]	Open-label RCT	Soy milk (12.5 g protein, 17.13 mg isoflavones) or soy milk + 1-h walking/day vs. control for 3 months.	FRAP	Postmenopausal women: 15 soy, 12 soy + walking, 10 control	Iranian	Improved antioxidant activity in soy arms.
Hanwell et al. 2009 [50]	Cross-over RCT	Single meal fish oil and/or 96 mg soy isoflavone vs placebo with 6h post-prandial evaluation each.	LOOH, Ox-LDL, ABTS	10 overweight or obese men	North-American	Not significant.
Hariri et al. 2015 [51]	Double-Blind RCT	200 mL/day probiotic soy milk or soy milk (control) for 8 weeks.	SOD, 8-OHdG	40 patients with T2DM (20 Intervention and 20 control)	Iranian	Higher SOD and lower 8-OHdG in the probiotic group but not in the control group.
Hematdar et al. 2018 [52]	Single-blind RCT	1 cup of soybeans, three days/week vs. legume or control groups for 8 weeks.	TBARS	Patients with T2DM: 21 soy, 20 legume, 23 control	Iranian	Not significant.
Heneman et al. 2007 [53]	Cross-over RCT	Single shake intake containing milk protein (placebo), 25 g soy protein with 107 mg isoflavones or soy with <4 mg isoflavones with 8h post-prandial evaluation each.	FRAP, ORAC, PCA-ORAC, Ox-LDL, Whole plasma oxidation	16 healthy adults	North-American	Not significant.
Hill et al. 2004 [54]	Double-blind RCT	39 g/day of soy protein (88 mg aglycone isoflavones) vs. 39 g/day whey protein (placebo), 4 weeks before moderate-intensity weight resistance exercise.	LOOH	Young adult men, recreationally trained: 9 treatment, 9 placebo	North-American	Lower exercise-induced oxidative stress in soy arm.
Jamilian et al. 2015 [55]	Double-Blind RCT	30 g/day textured soy protein (75 mg phytoestrogen) or animal protein (control) for 6 weeks.	FRAP, GPx, TBARS	Women with GDM: 34 soy, 34 control	Iranian	Improvement of biomarkers. After adjustment for baseline values, FRAP and GPx lost significance.
Jamilian et al. 2016 [56]	Double-Blind RCT	50 mg/day soy isoflavones vs. placebo for 12 weeks.	TBARS, GSH, FRAP	70 women with PCOS (35 intervention, 35 placebo)	Iranian	MDA and GSH improved in soy arm, FRAP values were not significantly different after adjustment for baseline.
Jenkins et al. 2000 [57]	Cross-over RCT	93% of animal protein replaced with soy and other vegetable protein (86 mg total isoflavone per 2000 Kcal) vs. LOV low-fat diet (no isoflavones, control) for 1-month each.	Ox-LDL	31 hyperlipidemic subjects	Canadian	Lower LDL oxidation.
Jenkins et al. 2000 [58]	Cross-over RCT	Soy flour added cereal/day (36 g protein, 168 mg isoflavones) vs. cereal control for 3 weeks each.	Ox-LDL	25 hyperlipidemic subjects	North-American	Lower LDL oxidation.
Jenkins et al. 2002 [59]	Cross-over RCT	50 g/day soy protein (73 mg isoflavones, treatment) vs. 52 g soy protein (10 mg isoflavones, placebo) vs low-fat dairy for 1 month (control).	Ox-LDL	37 hyperlipidemic men and postmenopausal women	North-American	Not significant. CONTRAST test showed significant negative percentage change of Ox-LDL in soy phase compared to control.
Karamali et al. 2018 [60]	RCT	30 g/day textured soy protein (75 mg phytoestrogen) vs. control for 8 weeks.	FRAP, GPx, TBARS	Patients with PCOS: 30 soy, 30 control	Iranian	Improvement of GPx (per time and group), and MDA (per time). FRAP was not significant. The correlations did not change after metabolic profile adjustments.
Kwak et al. 2013 [61]	Double-Blind RCT	4.5 g/day black soy peptides or casein (placebo) for 8 weeks.	PGF2α, TBARS, SOD	Patients with pre-hypertension: 45 soy, 46 placebo	Korean	Improved antioxidant capacity. A significant correlation between ACE and SOD.
Lee et al. 2006 [62]	Single-blind, Cross-over RCT	30 mL dark soy sauce or colorant (placebo) + 200g boiled rice with post-prandial 4 h evaluation each.	Serum and U PGF2α, U 8-OHdG	24 healthy adults	Singaporean	Plasma 8-isoprostane levels were lower to a greater extent in the soy phase. DNA damage and U 8-isoprostane were not significantly different.
Li et al. 2017 [63]	Double-blind RCT	80 mg/day isoflavones vs. placebo for 4 weeks.	SOD, PGF2α, TBARS, Nrf2	Patients with IHD: 100 treated, 100 placebo	Chinese	Improvement of oxidative biomarkers.
Miraghajani et al. 2012 [64]	Cross-over RCT	240 mL/day of soy milk vs cow’s milk for 4 weeks each.	TBARS	25 patients with T2DM and nephropathy	Iranian	Not significant.
Miraghajani et al. 2017 [65]	Double-blind RCT	200 mL/day probiotic soy milk or soy milk (control) for 8 weeks.	PGF2α, TBARS, GPx, GSH, GSR, GSSG, FRAP	Patients with T2DMKD: 20 probiotic, 20 control	Iranian	Higher values for GSH, lower GSSG, in the probiotic arm. The two arms provoked increased activity of GPx (higher for control) and GSR (higher for treatment). Other markers were not significant.
Mitchell et al. 1999 [66]	Open-label RCT	1 L/day soy milk, rice milk or cow’s milk for 4 weeks.	Comet Assay, FRAP	Healthy men: 4 soy, 3 rice, 3 cow’s	Scottish	Reduced DNA oxidative damage. No effect on FRAP or H2O2-induced DNA damage protection.
Mittal et al. 2014 [67]	Double-blind RCT	75 mg/day isoflavones vs. placebo for 12 weeks.	TBARS, SOD, GPx, CAT	Oophorectomized women: 17 treatment, 17 placebo	Indian	Not significant.
Nestel et al. 1997 [68]	Cross-over RCT	80 mg/day isoflavones or placebo for 5 weeks each	Ox-LDL	21 peri- and postmenopausal women	Australian	Not significant.
Nhan et al. 2005 [69]	Non-randomized Cross-over, CT	1 L soy milk with 113-207 mg/day isoflavones vs low isoflavone soy milk (<4.5 mg/day) for one menstrual cycle each.	U PGF2α	8 premenopausal women	North-American	No significant differences between phases. Age-adjusted Correlation between urinary 8-isoprostane change and isoflavone intake and excretion.
Oh et al. 2005 [70]	Single-arm, open-label trial	2 g/day of genistein combined polysaccharides (120 mg genistein, 57 mg daidzein) for 12 weeks.	GPx, CAT, PON	12 postmenopausal women with diabetic retinopathy	Korean	Increased GPx activity. Unchanged CAT and PON.
Önning et al. 1998 [71]	RCT	0.75 (M) 1 (W) L/day of soy or cow milk for 4 weeks.	ABTS	23 Healthy subjects (12 soy, 11 cow)	Swedish	Not significant.
Pusparini et al. 2013 [72]	Double-blind RCT	100 mg/day isoflavones vs. placebo for 12 months	TBARS	Postmenopausal women: 90 treated, 92 placebo	Indonesian	Reduced oxidative marker.
Reverri et al. 2015 [73]	Cross-over RCT	70 g soy nuts/day vs. control snack for 4 weeks each.	Ox-LDL	17 adults with cardiometabolic risk	North-American	Not significant.
Rossi et al. 2000 [74]	Single-Blind RCT	2 soy beverage/day (40 g protein, 44 mg genistein) vs. whey beverage (placebo) for 3 weeks and before and after a strenuous aerobic exercise bout.	ABTS, MPO, Uric acid	10 young males	North-American	Increased TAS in soy arm, reduced MPO and increased uric acid after exercise bout in soy arm.
Ryan-Borchers et al. 2006 [75]	Double-Blind RCT	706 mL/day Soy milk (71.6 mg isoflavones), 706 mL cow’s milk + 70 mg isoflavones or cow’s milk (placebo) for 16 weeks	PGF2α, 8-OHdG	Postmenopausal women: 18 Soy, 15 cow’s milk + isoflavones, 19 control	North-American	Similar reduced DNA damage marker in interventional arms. Not significant isoprostane variation.
Sedaghat et al. 2019 [76]	Single-blind RCT	60 g soy nut/day vs. control for 8 weeks.	FRAP	Patients with T2DM: 34 intervention, 34 control	Iranian	Improved antioxidant capacity.
Sen et al. 2012 [77]	Cross-over RCT	2 soy food servings/day (soy milk, soy nuts or tofu) with 50 mg aglycone isoflavones vs control for 6 months each.	U PGF2α	82 premenopausal women	Hawaiian	Oxidative markers had a little worsened in soy phase.
Serrano et al. 2017 [78]	Cross-over RCT	Postprandial response at 60 min after single intake each of 500 mL soy beverage vs. 500 mL glucose solution (placebo) or 500 mL water (control).	FRAP	29 healthy, non-smokers, young adults	Spanish	Higher antioxidant response.
Steinberg et al. 2003 [79]	Cross-over RCT	25 g/day of soy protein isolate with or without 107 mg of isoflavones or phytate for 6 weeks each.	Ox-LDL	28 healthy postmenopausal women	North-American	Not significant.
Swain et al. 2002 [80]	Double-Blind RCT	40 g SPI high-isoflavone (treatment) vs SPI low-isoflavone (placebo) and 40 g whey protein isolate (control) for 24 weeks.	ABTS	24 perimenopausal women: 4 treatment, 24 placebo, 21 control.	North-American	Not significant time-treatment differences. A positive correlation between TAS and soy intake in multiple regression analysis at 12 weeks.
Taniguchi-Fukatsu et al. 2012 [81]	Cross-over RCT	200 g/day Natto vs. non-viscous boiled soybeans (placebo) for two weeks each.	MDA-LDL	11 overweight adults with IGT	Japanese	Reduced oxidative biomarker.
Tikkanen et al. 1998 [82]	Single-arm, open-label trial	Two soy bar/day with a total of 38 mg isoflavones for 2 weeks.	Ox-LDL	6 healthy subjects	Finnish	Improved antioxidant effect.
Utarwuthipong et al. 2009 [83]	Cross-over RCT	20% of energy intake from soybean oil, rice bran oil, palm oil or 3:1 mixture of RBO/PO for 10 weeks each.	Ox-LDL	16 hypercholesterolemic women	Thai	Increased oxidation lag time in PO, RBO and RBO/PO but reduced in SBO.
Vega-López et al. 2005 [84]	Cross-over RCT	25 g/1000 kcal/day animal or soy protein (with or without 40 mg isoflavone/1000 kcal/day) for 42 days each.	TAP, Ox-LDL, U PGF2α, TBARS, Plasma protein carbonyls (on native and oxidized plasma)	42 hypercholesterolemic old adults	North-American	Only TAP and protein carbonyls (oxidized plasma) show amelioration in soy protein phases. MDA was higher in isoflavones diet.
Wiseman et al. 2000 [85]	Cross-over RCT	One textured soy protein burger/day with 15 g protein and 56 mg isoflavones vs. burger without isoflavones (alcohol extracted) as a placebo for 17 days each.	PGF2α, Ox-LDL, TBARS	24 healthy adults	British	8-isoprostane and Ox-LDL were improved in intervention, TBARS was not significant.
Zemel et al. 2010 [86]	Cross-over RCT	3 smoothies/day diary-based or soy-based (control) for 28 days each.	TBARS, PGF2α	20 healthy adults (including 10 overweight)	North-American	Improved antioxidant ability in the diary phase. No significant differences for soy treatment.

Abbreviations: 2,2’-azino-di-(3-ethylbenzthiazoline sulfonate) (ABTS); 5-hydroxymethyl-2’deoxyuridine (5-OHmdU); 8-hydroxy-2’-deoxyguanosine (8-OHdG); 8-iso-prostaglandin F2α (PGF2α); Advanced oxidation protein products (AOPP); Auto-antibodies for oxidized LDL (Ox-LDL Ab); Catalase (CAT); Copper (Cu); Cyclooxygenase-2 (COX-2); Ferric-reducing antioxidant power (FRAP); Ferrous oxidation xylenol orange, version 1 (FOX 1); Glutathione peroxidase (GPx); Glutathione reductase (GSR); Lipid hydroperoxides (LOOH); Lipophilic aldehydes and related carbonyl compounds (LARC); Malondialdehyde-modified low density lipoprotein (MDA-LDL); Myeloperoxidase (MPO); Nuclear factor erythroid 2-related factor 2 (Nrf2); Oxidized glutathione (GSSG); Oxidized LDL (Ox-LDL) ; Oxygen radical absorbance capacity (ORAC); Paraoxonase (PON) ; Perchloric-acid-treated oxygen radical absorbance capacity (PCA-ORAC); Plasma total antioxidant performance (TAP); Reduced glutathione (GSH); Superoxide dismutase (SOD); Thiobarbituric acid reactive substances (TBARS); Total peroxyl radical-tapping anti-oxidant potential (TRAP); Urinary 8-hydroxy-2’-deoxyguanosine (U 8-OHdG); Urinary 8-iso-prostaglandin F2α (U PGF2α).

**Table 2:** Biomarkers for antioxidant capacity and oxidative status.

Name	Description
2,2’-azino-di-(3-ethylbenzthiazoline sulfonate) (ABTS)	Total Antioxidant Status (TAS) determined with a colorimetric method based on the inhibition of ABTS (2,2’-azino-di-(3-ethylbenzthiazoline sulfonate)) oxidation by a peroxidase. Values are expressed in Trolox Equivalent antioxidant capacity (TEAC) [87].
5-hydroxymethyl-2’deoxyuridine (5-OHmdU)	It is a modified DNA by-product generated by thymine oxidation, that represents a marker of oxidative damage. After isolation and purification of nucleic acids, 5-OHmdU is detected through GC-MS.
8-hydroxy-2’-deoxyguanosine (8-OHdG)	It is a common marker of oxidative DNA damage involved in cancer genesis. It can be measured in various biological samples with several methods including HPLC, GC-MS, or ELISA [88].
8-iso-prostaglandin F2α (PGF2α)	The most abundant arachidonic acid oxidation by-product. It can be quantified in blood or urine samples by an enzyme immunoassay method (EIA) or GC-MS [89].
Advanced oxidation protein products (AOPP)	Oxidative by-products of plasma proteins, usually in uremic patients, detected with spectrophotometric assay [90].
Catalase (CAT)	An antioxidant enzyme that catalyzes the conversion of hydrogen peroxide into water and oxygen. The enzyme activity is detected spectrophotometrically.
Comet assay	It is a single-cell microgel electrophoresis (SCGE) assay to detect the frequency of DNA breaks or DNA oxidized bases (with a modification of the original assay using an endonuclease to break DNA at sites of oxidized bases) [91].
Cyclooxygenase-2 (COX-2)	It is an enzyme involved in prostanoids metabolism by the conversion of arachidonic acid. Its oxidative action is of interest in oxidative stress and inflammation. It is usually detected using commercial ELISA kits.
Ferric-reducing antioxidant power (FRAP)	Total antioxidant capacity (TAC) determined by a colorimetric assay using ferric-reducing antioxidant power [92].
Ferrous oxidation xylenol orange, version 1 (FOX1)	An assay to detect plasma lipid hydroperoxides through ferrous ion oxidation with the ferric ion indicator xylenol orange reagent [93].
Glutathione peroxidase (GPx)	An antioxidant enzyme that catalyzes the reduction of lipid hydroperoxides and hydrogen peroxide into corresponding alcohols and water, respectively. Its activity is detected spectrophotometrically.
Glutathione reductase (GSR)	An enzyme that catalyzes the reconversion of GSSG into GSH using NADPH. Its activity is detected spectrophotometrically.
Lipophilic aldehydes and related carbonyl compounds (LARC) and lipid hydroperoxides (LOOH)	Aldehydes e carbonyl compounds are intermediate and secondary products of oxidation in lipid peroxidation pathways, respectively [94]. Extracted compounds from bodily fluids are detected by commercial spectrophotometric assays or HPLC separation after 2,4-Dinitrophenylhydrazine (DNPH) derivatives compound formation.
Malondialdehyde-modified LDL (MDA-LDL)	A by-product of oxidized LDL [95]. Compounds are detected by ELISA.
Myeloperoxidase (MPO)	A prooxidant enzyme expressed primarily by neutrophil granulocytes as an antimicrobial defense strategy. Its action may also cause oxidative damage to the host. It is usually detected by ELISA.
Nuclear factor erythroid 2-related factor 2 (Nrf2)	It is an antioxidant related molecule acting as a transcription factor regulating the cellular resistance to oxidative stress [96]. It is detected with a semi-quantitative colorimetric assay kit using a nuclear extract.
Oxidized glutathione (GSSG)	It is the oxidized, disulfide form of glutathione generated after the reaction of GSH with reactive oxygen species (ROS). In healthy conditions, only a minor part of glutathione is in the GSSG form. A lower GSH/GSSG ratio show the oxidative stress status. It is usually detected using commercial ELISA kits.
Oxidized LDL (Ox-LDL) and auto-antibodies for oxidized LDL (Ox-LDL Ab)	An ex-vivo method based on the formation of conjugated dienes catalyzed by incubation with Cu^2+^ or myeloperoxidase as prooxidants, on isolated LDL from plasma [97]. Ox-LDL can be detected also dosing Ox-LDL auto-antibodies.
Oxygen radical absorbance capacity (ORAC)	Measurement of the overall plasma capacity to scavenge oxygen radicals using a peroxyl generator, such as 2,2′-Azobis(2-amidino-propane) dihydrochloride (AAPH), and a fluorescent probe [98].
Paraoxonase (PON)	Antioxidant enzyme preventing lipoprotein oxidation by lactonase and esterase action. The enzyme activity in the solution is measured spectrophotometrically.
Plasma protein carbonyls	Protein oxidation by-products generally detected by a colorimetric ELISA assay.
Plasma total antioxidant performance (TAP)	Fluorometric assay performed in a multilabel counter using dichlorodihydrofluorescein (DCFH), a probe for oxidative activity [99].
Reduced glutathione (GSH)	It is a molecule that reacts with free radicals and peroxides quenching oxidative stress generated by these molecules. It is a cofactor for GPx. It is usually detected using commercial ELISA kits.
Superoxide dismutase (SOD)	An antioxidant enzyme that catalyzes the dismutation of superoxide radicals into hydrogen peroxide and molecular oxygen. It is usually detected using commercial ELISA kits.
Thiobarbituric acid reactive substances (TBARS)	Lipid peroxidation quantification based on a spectrophotometrical or HPLC assay for the quantification malondialdehyde (MDA) with thiobarbituric acid reactive substances through the formation of MDA-TBA adduct [100].
Total peroxyl radical-tapping anti-oxidant potential (TRAP)	Measurement of the radical-trapping capacity of whole plasma using a free-radical probe [101].
Uric acid	It is a metabolite of purine nucleotides oxidative breakdown. Its role in antioxidant capacity has been speculated. It is detected by quantitative colorimetric or fluorometric enzymatic kits.
Whole plasma oxidation	Assy based on conjugated dienes formation in whole plasma by Cu^2+^ and detection by a spectrophotometer [102].
